# Microtremor Recording Surveys to Study the Effects of Seasonally Frozen Soil on Site Response

**DOI:** 10.3390/s23125573

**Published:** 2023-06-14

**Authors:** Shengyang Chen, Jie Lei, Ya Li

**Affiliations:** 1 College of Civil Engineering, Tongji University, Shanghai 200092, China; chenshengyang@tongji.edu.cn (S.C.); 1911671@tongji.edu.cn (J.L.); 2 Department of Civil Engineering, Shanghai Normal University, Shanghai 201418, China

**Keywords:** microtremor recording, seasonally frozen soil, HVSR method, predominant frequency, amplification factor, cover thickness

## Abstract

Microtremor recording tests using an accelerometer were carried out in this paper with the aim of characterizing the effects of seasonally frozen soil on the seismic site response, including the two-direction microtremor spectrum, site predominant frequency, and site amplification factor. The study selected eight typical seasonal permafrost sites in China for site microtremor measurements during both summer and winter seasons. Based on the recorded data, the horizontal and vertical components of the microtremor spectrum, HVSR curves, site predominant frequency, and site amplification factor were calculated. The results showed that seasonally frozen soil increased the predominant frequency of the horizontal component of the microtremor spectrum, while the effect on the vertical component was less noticeable. It indicates that the frozen soil layer has a significant impact on the propagation path and energy dissipation of seismic waves in the horizontal direction. Furthermore, the peak values of the horizontal and vertical components of the microtremor spectrum decreased by 30% and 23%, respectively, due to the presence of seasonally frozen soil. The predominant frequency of the site increased by a maximum of 35% and a minimum of 2.8%, while the amplification factor decreased by a maximum of 38% and a minimum of 11%. Additionally, a relationship between the increased site predominant frequency and the cover thickness was proposed.

## 1. Introduction

Seasonally frozen soil is a solid freezing layer that forms on the ground during the winter season and is widely distributed in many parts of the world, including the Northeast China, Alaska, and other regions [1,2]. The change in stiffness inevitably affects the dynamic properties of the site, such as how the Young’s modulus of frozen soil is tens to hundreds of times higher than that of unfrozen soil [3], leading to changes in soil dynamic properties that may impact seismic response. Therefore, understanding the impact of seasonally frozen soil on seismic site response is crucial for designing transportation infrastructure in cold regions.

In recent years, several researchers have investigated the dynamic properties of frozen soil using laboratory and field measures [4,5,6]. For example, LeBlanc [4] found that the P-wave and S-wave velocities in permafrost vary from 2400 to 3200 m/s and from 900 to 1750 m/s, respectively, for a temperature range between −0.2 and −2.0 °C. Additionally, Young’s modulus ranges from 2.15 to 13.65 GPa, and the shear modulus varies from 1.00 to 4.75 GPa over the same range of temperatures. Xu [5] examined the effect of seasonally frozen soil on the site seismic response and found that the spectral response of ground motions decreases as the thickness of the seasonally frozen soil increases, but was insensitive to the shear wave velocity of the seasonally frozen soil. Previous studies (Singh and Donovan 1977 [7]; Qi et al. 2006 [8]) have also demonstrated that the presence of the frozen surface layer affects ground motion characteristics and reduces the observed surface acceleration. However, current design codes such as AASHTO [9] have not provided any guidance regarding the effects of frozen soil.

While laboratory and field measures are the primary methods for investigating the effects of seasonally frozen soil on site seismic response, they are often prohibitively costly and difficult to deploy in urban areas [10]. Given these limitations, Qi [8] employed the Fourier amplitude spectra method (a technique of the microtremor method that utilizes one-direction microtremor recording Fourier spectra) to examine the variations in site predominant frequency between summer and winter in Zhangye, China. The results showed that the frozen soil layer, with a thickness of approximately 1 m, led to a 1.9 Hz increase in the predominant frequency of the noise Fourier spectrum.

One limitation of the one-direction microtremor recording Fourier spectra method is that it presupposes the seismic noise source to be white noise, which is not always true in real-world scenarios. Consequently, this approach can lead to inaccuracies in determining the predominant frequency of the microtremor spectrum, making it challenging to estimate the site amplification factor [11]. On the other hand, numerous studies have demonstrated that the microtremor horizontal-to-vertical spectral ratio method (HVSR) effectively eliminates the influence of the vibration source on the site dynamics analysis, resulting in predominant frequencies that are more representative of the actual site frequencies [12,13,14,15,16,17,18,19]. As a consequence, many recent studies have employed the microtremor HVSR method to determine the site characteristic parameters. However, the effects of seasonally frozen soil on the seismic site response through the use of the HVSR method remains limited in published research.

In seasonally frozen regions, the existence of a frozen layer induces seasonal fluctuations in the mechanical properties of the ground. This variation exerts a substantial influence on the ground motion characteristics of specific sites and, as a result, on the methods employed in seismic design for buildings. The research effort presented in this paper focuses on the effects of seasonally frozen soil on the site seismic response using the microtremor HVSR method. Eight sites in China were selected to represent typical sites with seasonally frozen soils. After that, microtremor measurements were carried out for the eight sites in summer and winter, respectively. The experimental details discussed in this paper may be of interest to researchers in this field. Moreover, the results would expand the application of the HVSR in the urban region and provide references for geotechnical engineering and seismic hazard assessment in areas with frozen soils.

## 2. General Geology

**Figure 1:**
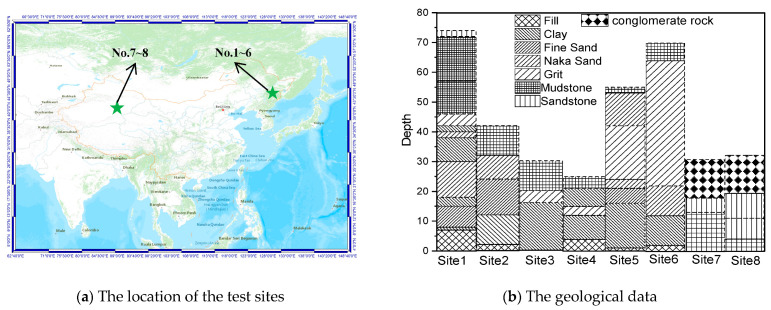
The location of the test sites and the geological data.

Eight sites in the northeast and northwest regions of China (Figure 1a) were selected for this study. The soil in these areas has a surface layer that ranges from 1.5 to 2 m in thickness and undergoes annual freezing and thawing. The geological data (collected during the summer) of these eight sites are presented in Figure 1b, and the site characteristic parameters such as *V_s_*_20_ (average shear wave velocities to a depth of 20 m), *V_s_*_30_ (average shear wave velocities to a depth of 30 m), and the thickness of sediments (*h*) are listed in Table 1. Site No.1 to No.6 are located in Northeast China and are comprised of a main component of clay, while the remaining two sites in Northwest China have a main component of gravel. The soil in these sites is composed of three layers in general, with 0 to 7 m of the top layer filled.

The *h* (thickness of sediments) of the eight sites varied between 6 and 75 m during the summer. Specifically, two sites in Northwest China had *h* values of 6 and 11 m, respectively. The *V_s_*_20_ ranged from 165 m/s to 318 m/s, with two sites in Northwest China having *V_s_*_20_ values of 318 m/s and 317 m/s, respectively. The *V_s_*_30_ ranged from 194 m/s to 558 m/s, with two sites in Northwest China having *V_s_*_30_ values of 558 m/s and 520 m/s, respectively.

## 3. Microtremor Data

Microtremor measurements were carried out at eight sites in July and January, respectively. The average temperature in the selected sites during July was 25 °C, the highest monthly average temperature in a year, and the frozen soil had completely melted. Conversely, in January, the temperature reached its lowest point of −25 °C.

**Figure 2:**
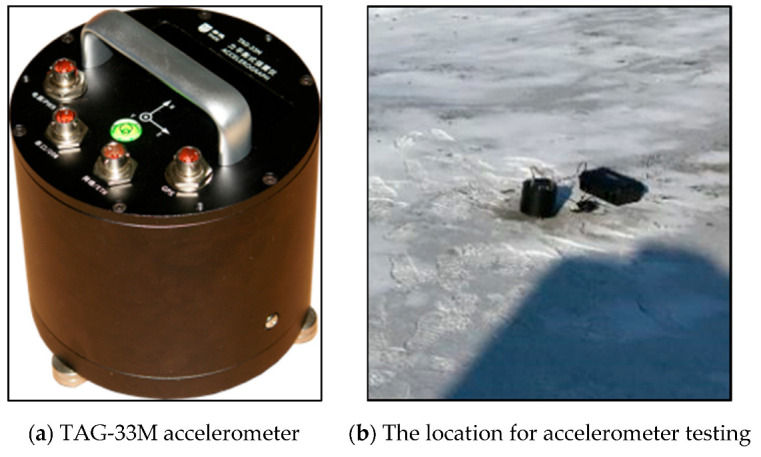
TAG-33M accelerometer.

The TAG-33M accelerometer (The manufacturer is TAIDE Company, located in Zhuhai City, Shenzhen, China), which integrates a sensor and a data collector, was used for measuring microtremors in three directions. This accelerometer is ideal for field measurements and other applications, as shown in Figure 2. It features an antioxidation aluminum casing, waterproof connectors, and stainless-steel leveling and fixing screws, which allow it to function under challenging field conditions. The compact device is simple to install and troubleshoot. It has no physical buttons on its surface and is controlled using an infrared remote control to ensure optimal sealing and waterproofing. The TAG-33M utilizes digital output, enabling easy networking through the internet or a serial port for robust earthquake monitoring. This makes it ideal for mobile field observations and other applications. Additionally, the accelerometer has a sampling frequency range of 0.001 Hz to 200 Hz and is capable of operating at temperatures ranging from −40 °C to 60 °C, making it suitable for the low-temperature conditions of the winter sites.

**Figure 3:**
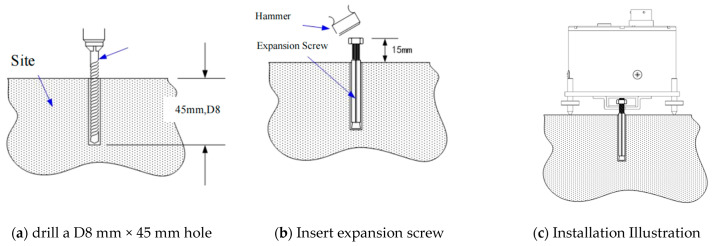
The installation procedure for TAG-33M.

The installation procedure for recording ground microtremors using the TAG-33M accelerometer is as follows:

(a) After selecting the appropriate installation location, use a compass to mark the true north and east directions on the surface; then, drill a D8 mm × 45 mm hole at the installation location using an impact drill (Figure 3a).
(b) Insert an M6 × 60 expansion screw into the hole and tighten it with a hammer (Figure 3b).
(c) Loosen the nut and place the accelerometer in the center position; then, tighten the nut to secure it onto the accelerometer’s clip.
(d) Adjust the orientation of the accelerometer, and then adjust the leveling screws until the bubble is centered (Figure 3c).

**Figure 4:**
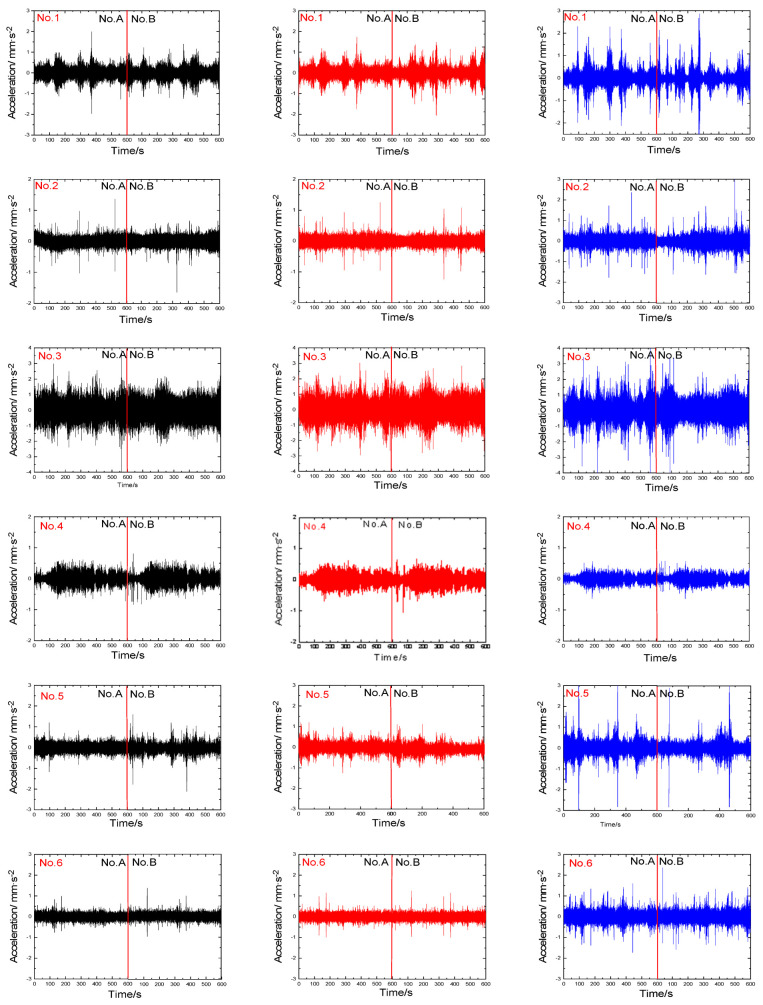

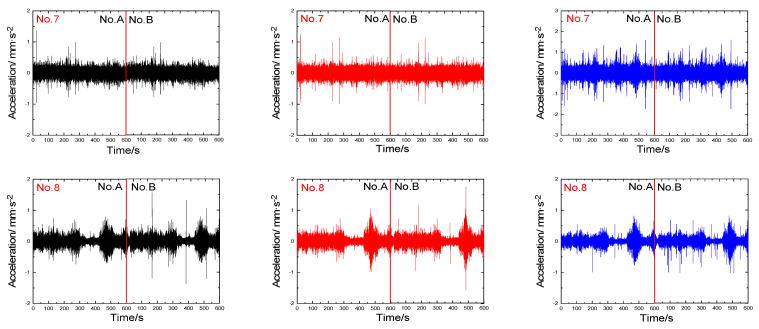
The unfiltered noise recordings were obtained in January. (The recordings are depicted in black, red, and blue, representing the north−south (NS), east−west (EW), and up−down (UD) directions, respectively).

**Figure 5:**
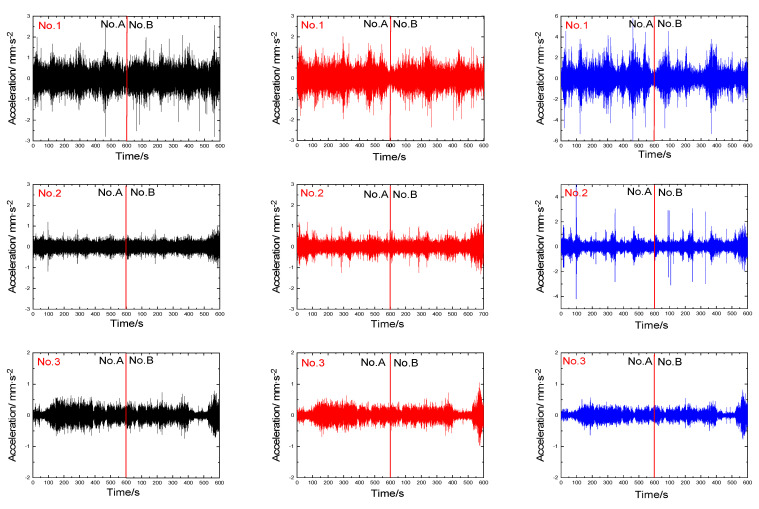

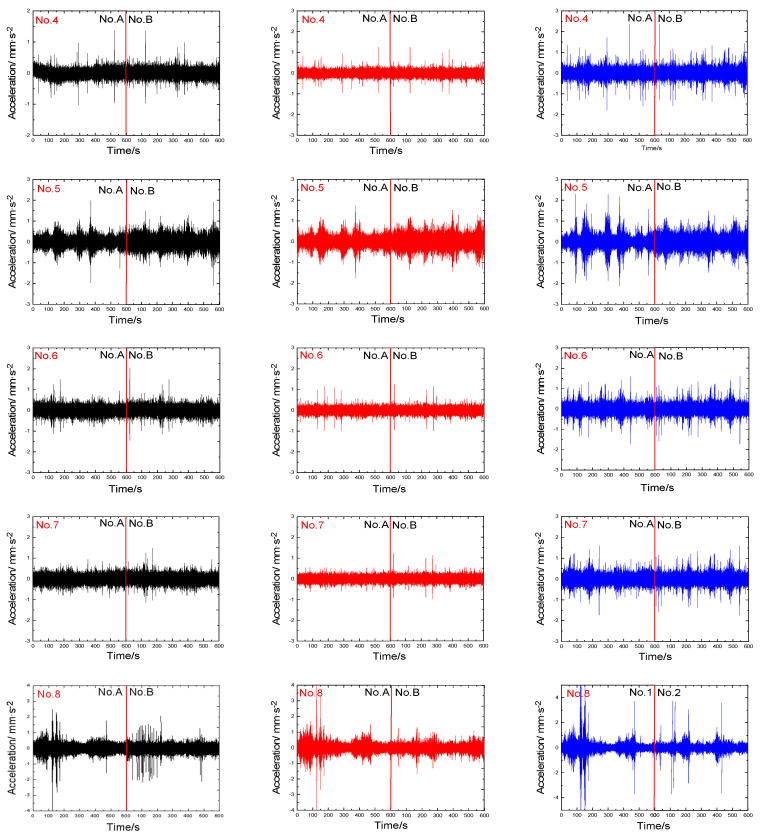
The unfiltered noise recordings were obtained in July. (The recordings are depicted in black, red, and blue, representing the north−south (NS), east−west (EW), and up−down (UD) directions, respectively).

To ensure reliable microtremor recordings, two instruments (abbreviated to No.A and No.B, respectively) were placed on the same borehole. Each recording lasted 10 min, with a sampling rate of 100 Hz. The unfiltered noise recordings for two instruments (including three directions: up–down, north–south, and east–west) are displayed in Figure 4 and Figure 5. 

## 4. Microtremor Characteristics

### 4.1. Calculate Microtremor Spectrum

**Figure 6:**
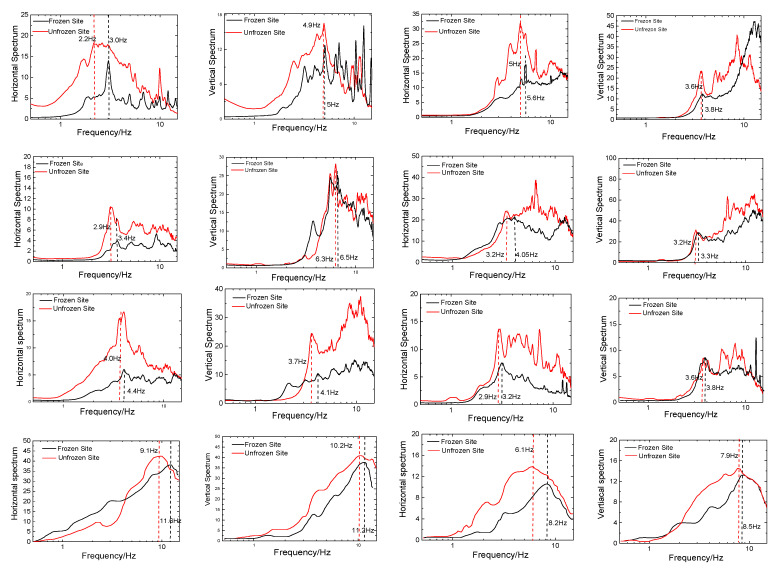
Horizontal and vertical microtremor spectra for eight sites in summer (red) and winter (black) (Site No.1 is top left, Site No. 8 is bottom right).

**Figure 7:**
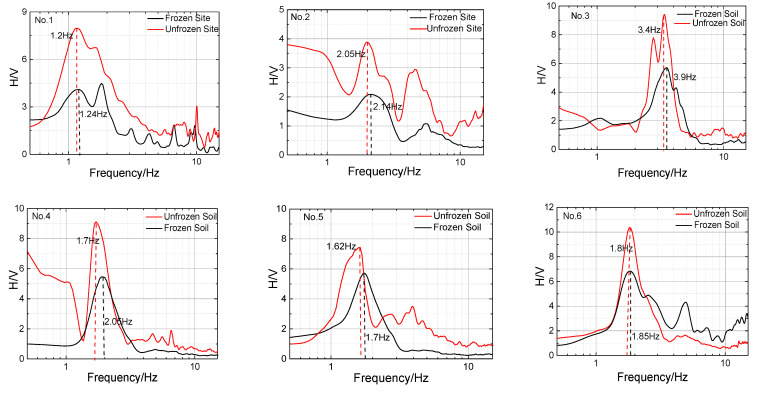

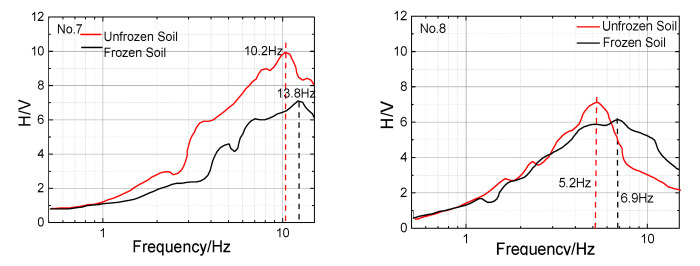
HVSR curve for eight sites in in summer (red) and winter (black).

Following the Site Effects Assessment using Ambient Excitations (SESAME) criteria [20], the signals (Figure 4 and Figure 5) relative to each window were detrended (Using J-SESAME 1.08 software, transients were detected while efforts were made to avoid them. The procedure for detecting transients involves comparing the short-term average (STA), which represents the average signal amplitude over a short time period “tsta” (typically ranging from 0.5 to 2.0 s), with the long-term average (LTA), which represents the average signal amplitude over a much longer time period “tlta” (typically spanning several tens of seconds). The objective is to maintain the STA/LTA ratio below 2 for a sufficient duration. Additionally, the selection of windows in the ambient vibration data aims to exclude those with abnormally low amplitudes. To achieve this, a minimum threshold value of 0.1 is introduced to ensure that the signal within the selected ambient vibration windows does not fall below this threshold.), baseline-corrected, tapered, and band-pass-filtered between 0.1 and 25 Hz. Then, the fast Fourier transform was applied to the three signal components for each 20.48 s long window (Equation (1)). Afterward, Equation (2) was employed to calculate the horizontal spectrum, and the HVSR curves were determined using the Konno and Ohmachi [21] method, as described by Equation (3). Although the sampling frequency was 100 samples per second, the amplitude spectral range displayed was 0.1 to 25 Hz, which is typically considered appropriate for studies in seismic microzoning and earthquake engineering. The results of the horizontal spectrum, vertical spectrum, and HVSR curves for eight sites in winter and summer are presented in Figure 6 and Figure 7, respectively.

(1) $$F_{\left(NS\backslash EW\backslash UD\right)}=\sum_{i=1}^{n}F_{i(NS\backslash EW\backslash UD)}/n$$

(2) $$F_{H}=\sqrt{\left(F_{NS}^{2}+F_{EW}^{2}\right)}$$

(3) $$HVSR=\sum_{i=1}^{n}\sqrt{\frac{F_{i(EW)}^{2}+F_{i(NS)}^{2}}{2F_{i(UD)}^{2}}}/n$$

in which *n* is the number of the time window, *n* = 1200 s/20.48 s, and $F_{i(NS\backslash EW\backslash UD)}$ is the *i*-th time window spectrum for NS, EW, and UD directions.

To calculate the amplification factor of the eight sites in winter and summer, the estimation method for the site amplification factor proposed by Nakamura [11] was used. A brief overview of the method is provided below. Nakamura posited that if the surface layer consists of a single layer, the vibration at the base ground boundary (*B*) can be estimated from the vibration at the ground surface (*S*) using Equation (4). *h*/*Vs* (*h* and *Vs* are the depth and velocity of shear wave propagation for the ground surface, respectively) in Equation (4) can be derived from Equation (5).

(4) $$B(t)=\{S(t+h/V_{s})+S(t-h/V_{s}\}$$

(5) $$h/V_{s}=1/(4f)$$

**Figure 8:**
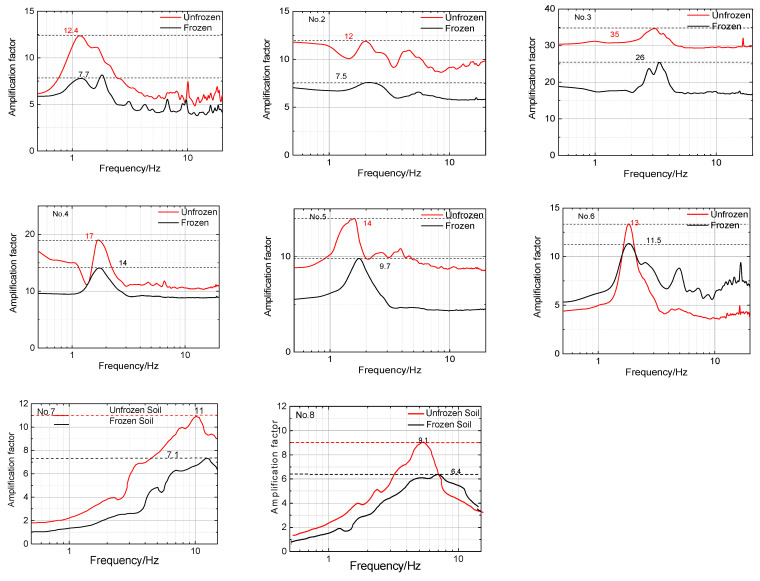
T(*f*) curves for eight sites in summer (red) and winter (black). (Dashed lines indicate peak values).

The transfer spectrum can be calculated as the spectral ratio between the ground surface and the basement; then, it is possible to estimate the amplification factor at the predominant frequency of T(*f*) (calculated by Equation (6)); the T(f) for eight sites is listed in Figure 8.

(6) T(f)=F[S(t)](f)/F[B(t)](f)

Here, *F*[*] is Fourier transfer.

### 4.2. Horizontal and Vertical Microtremor Spectrum

Figure 6 displays the microtremor recording of the two-component (horizontal and vertical) Fourier spectrum in winter and summer for the eight sites; the predominant frequency and peak value of the corresponding Fourier spectra are presented in Table 2. The results reveal that the seasonally frozen soil had a significant impact on the horizontal direction of the spectrum curves, but had a minor effect on the vertical direction. As demonstrated in Figure 5 and Table 2, the findings can be succinctly summarized as follows:

(a) The seasonally frozen soil increased the predominant frequency of both the horizontal and vertical components of the microtremor recording Fourier spectrum for the test sites. The average increase in the predominant frequency of the horizontal component was 20.8%, while that of the vertical component was 6%. The difference in the variation in the predominant frequency between the horizontal and vertical components was significant, indicating that the seasonally frozen soil primarily affected the predominant frequency of the horizontal component of the microtremor recording Fourier spectrum, with little impact on the vertical component.
(b) The seasonally frozen soil reduced the peak values of both the horizontal and vertical components of the microtremor recording Fourier spectrum for the test sites. The average decrease was 30% for the horizontal component and 23% for the vertical component, with no significant difference between the two. This indicates that the seasonally frozen soil layer had a similar impact on the peak values of the horizontal and vertical components of the microtremor recording Fourier spectrum.
(c) The thickness of the sediments and ground types had a significant impact on the influence of the seasonally frozen soil on the predominant frequency of the microtremor recording Fourier spectrum at the test sites. Specifically, at six of the test sites located in Northeast China with cover thicknesses greater than 20 m (class C), the predominant frequency of the horizontal and vertical components of the microtremor recording Fourier spectrum increased, on average, by 0.58 Hz and 0.2 Hz, respectively. However, for the remaining two test sites located in the Gobi sand with cover thicknesses less than 20 m (class B), the predominant frequency of the horizontal and vertical components of the microtremor recording Fourier spectrum increased, on average, by 2.3 Hz and 0.8 Hz.

Ibs-von Seth [22] showed that the frequency of resonance of the soil layer (estimated from the peak in the HVSR curve) is closely related to H through Equation (7).

(7) $$h=af_{r}{}^{b}$$

Therefore, Equations (8) and (9) can be derived, as shown below:

(8) $$f_{r}=ch^{d}$$

(9) $$\Delta f_{r}=eh^{g}$$

Using the $\Delta f_{m}$ (listed in Table 2) and *h* obtained from borehole data, the nonlinear regression fit of Equation (9) is performed, resulting in the derivation of Equations (10) and (11).

(10) $$\Delta f_{m/H}=10.45h^{-0.77}$$

(11) $$\Delta f_{m/V}=4.73h^{-0.88}$$

**Figure 9:**
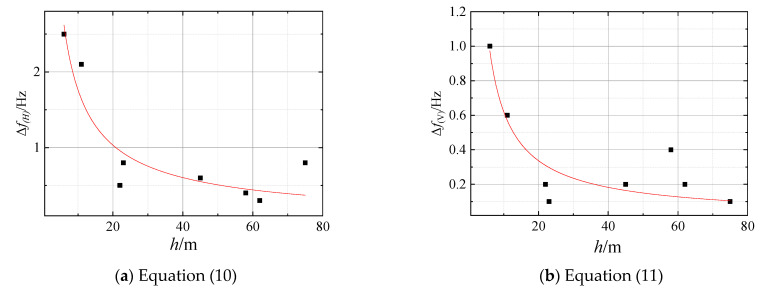
The relation between *h* and $\Delta f_{m}$. (Scatter points represent test data, the curve represents the fitted equation).

Equations (10) and (11) illustrate the correlation between the increase in the predominant frequency of the horizontal microtremor recording Fourier spectrum and cover thickness, as well as the correlation between the increase in the predominant frequency of the horizontal microtremor recording Fourier spectrum and cover thickness (Figure 9). The Adjust *R*^2^ of Equation (10) was 0.839, which was greater than the critical value of $R{(8-2)}_{0.01}=0.834$ (critical table of correlation coefficients). On the other hand, the Adjust *R*^2^ of Equation (11) was 0.774, which is less than 0.834. These results suggest a strong correlation between $\Delta f_{m}$ and cover thickness (h). The correlation between $\Delta f_{m}$ and *h* is better for the horizontal component of the microtremor recording Fourier spectrum compared to the vertical component.

Here, H is the horizontal microtremor recording Fourier spectrum, and V is the vertical microtremor recording Fourier spectrum. 

### 4.3. Site Predominant Frequency

**Figure 10:**
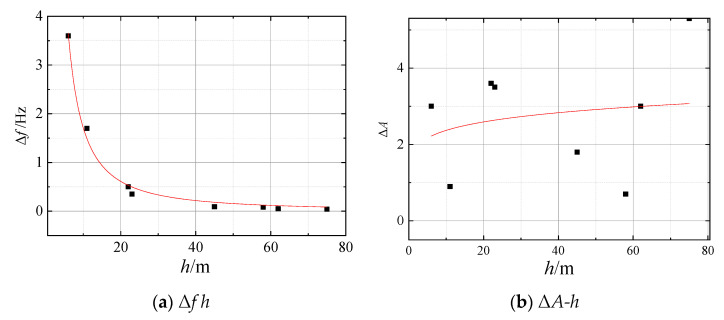
The relation between *h* with Δ*f* and Δ*A.* (Scatter points represent test data, the curve represents the fitted equation).

The predominant frequency of the test sites (estimated from the peak in the HVSR curve) is listed in Table 3; the following conclusions were drawn from the data presented in Table 3:

(a) The predominant frequency of all test sites during the freezing period was found to be higher than that during the nonfreezing period, with the highest increase reaching 35% and the lowest increase at 2.8%. This increase can be attributed to the effect of the seasonally frozen soil on increasing the stiffness of the site.
(b) For the sites with *h* greater than 40 m, the predominant frequency increment (Δ*f*) was less than 0.1 Hz. Conversely, for the remaining sites with *h* less than 40 m, the Δ*f* was, on average, 1.5 Hz. Notably, the increase in the predominant frequency was particularly significant for the site with *h* less than 20 m, suggesting a correlation between *h* and Δ*f*.
(c) The correlation between Δ*f* and *h* is examined using Equation (9) and is presented in Figure 10a. Equation (11) is the fitted relationship between Δ*f* and *h*, with an adjusted *R*^2^ value of 0.99. This value is higher than *R*(8−2)_0.001_ = 0.925, suggesting that the correlation is statistically significant.

In summary, the results of the study show that the seasonally frozen soil had a significant impact on the predominant frequency of the site and that this impact was inversely proportional to the thickness of the sediments; Δ*f* can be estimated using Equation (12).

(12) $$\Delta f=52.3h^{-1.485}$$

### 4.4. Site Amplification Factor

The T(*f*) curves and amplification factor of eight sites in different seasons are presented in Figure 8 and Table 4, respectively. The following conclusions were made based on the data shown in these two figures and table:

(a) The site amplification factor for all test sites was higher during the nonfreezing period than during the freezing period. The maximum difference was 38% and the minimum difference was 11%. This is likely due to the frozen surface layer dissipating wave motion energy and reducing the amplitude of the site compared to an unfrozen site.
(b) The amplification factor of the test sites with a soil surface during the freezing period was significantly lower than during the nonfreezing period, with an average decrease of 33%. However, for the test sites with continuous concrete and asphalt surfaces, the decrease in the amplification factor was relatively small, with an average decrease of 14%. This suggests that the surface layer environment plays a crucial role in the variation in the amplification factor between frozen and unfrozen sites.
(c) The correlation between *h* and the reduction in the site amplification factor (∆*A*) was investigated using Equation (8), and the fitting curve is plotted in Figure 10b. However, the *R*^2^ value of the fitting formula was found to be −0.124, indicating the lack of a significant correlation between *h* and ∆*A*.

## 5. Discussion

In Section 4.2, we studied the variation in the predominant frequency in the horizontal and vertical direction Fourier spectra under frozen soil conditions; the results showed that the impact of frozen soil was predominantly evident in the horizontal direction Fourier spectrum. 

A subsequent study found that the *f_H_* and *f* decreases as *h* increases; the Adjust *R*^2^ of Equations (10) and (12) were 0.834 and 0.99. It indicates that the correlation between *f* and *h* is stronger than the correlation between *f_H_* and *h*. It may contribute to the fact that the microtremor recording in one direction is susceptible to interference from environmental noise; the HVSR method can more effectively eliminate the impact of such noise compared to recording in only one direction.

Test sites No. 3 and No. 4 had similar soil layer composition and cover thickness, but ∆*f* values of 0.5 Hz and 0.35 Hz, respectively, and a relative deviation of 43%. Likewise, test sites No. 5 and No. 6 also had comparable soil layer composition and cover thickness, with ∆*f* values of 0.08 Hz and 0.05 Hz, respectively, and a relative deviation of 60%. The different changes in the site predominant frequency for the similar site characteristics may be attributed to the ground surface environment; the frozen soil layer resulted in a smaller increase in stiffness for the hard surface environment site compared to the soft surface environment site.

The variation in ∆A appears to be linked to the type of surface environment. For example, in test sites with soil surfaces, the average value of ∆A was 33%. However, in test sites with continuous concrete and asphalt surfaces, the ∆A had an average value of 14%. Additionally, there was no notable correlation found between h and ∆A.

**Figure 11:**
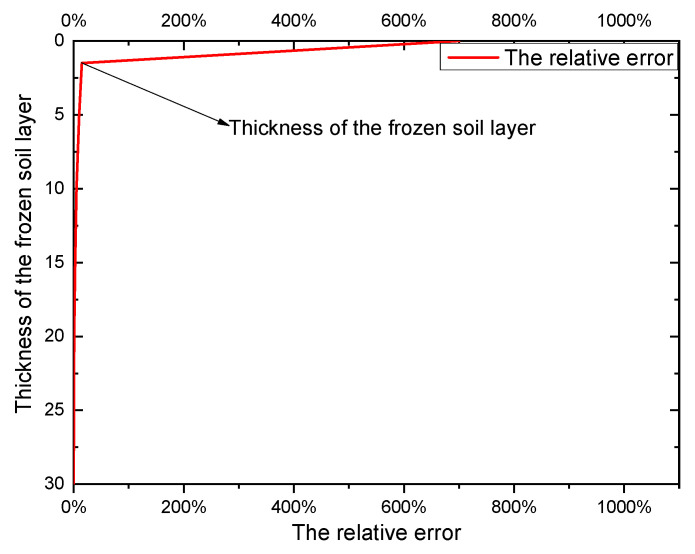
The relative error in shear wave velocity between the frozen and unfrozen soil layers.

The study by Cox B R [23] demonstrated that the presence of frozen soil increases the shear wave velocity of the ground, and the relative errors in shear wave velocity between frozen and unfrozen soil layers was calculated and presented in Figure 11. Based on the shear wave velocities of each soil layer obtained from drilling, the equivalent shear wave velocity of the unfrozen soil period was calculated using Equation (13). Then, assuming a frozen soil layer thickness of 1.75 m (the average of the maximum and minimum thicknesses), and considering that the shear wave velocity in the frozen soil layer was 100% higher than that in the unfrozen soil layer based on Figure 1, the equivalent shear wave velocity of the frozen soil period was calculated by incorporating the shear wave velocity in the frozen soil layer and using Equation (13). The results are listed in Table 5.

The frozen soil resulted in a 10% increase, on average, in the equivalent shear wave velocity across the eight sites. As indicated by Equation (5), the site predominant frequency (*f*) was positively correlated with the equivalent shear wave velocity. Therefore, *f* increases with the presence of frozen soil layers. We assume that these eight sites represent the majority of seasonal frozen soil sites. Therefore, for D-class sites with equivalent shear wave velocities greater than 162 (=180 − 180 × 10%) m/s, C-class sites with velocities greater than 270 (=300 − 300 × 10%) m/s, and B-class sites with velocities greater than 720 (=800 − 800 × 10%) m/s, it is recommended to increase the seismic design level by one grade during the freezing period.

(13) $${V_{S}}_{30}=30/\sum_{i=1}^{n}d_{i}/v_{i}$$

in which *d_i_* is the thickness of the *i*-th layer, and *v_i_* is the shear wave velocity of the *i*-th layer.

## 6. Conclusions

A comprehensive investigation of the effects of seasonally frozen soil on site characteristic parameters was carried out and presented in this paper. Eight sites with frozen soil layer thicknesses ranging from 1.5 m to 2.0 m in China were selected to represent typical sites with seasonally frozen soils. After that, microtremor measurements were carried out for the selected sites. For those sites, the conclusions can be summarized as follows:

(a) The seasonally frozen soil primarily affected the predominant frequency of the horizontal component of the microtremor recording Fourier spectrum, with little impact on the vertical component.
(b) The seasonally frozen soil reduced the peak values of both the horizontal and vertical components of the microtremor recording Fourier spectrum. For the test sites, the average decrease was 30% for the horizontal component and 23% for the vertical component. The seasonally frozen soil layer had a similar impact on the peak values of the horizontal and vertical components of the microtremor recording Fourier spectrum.
(c) The predominant frequency of all the test sites during the freezing period was found to be higher than that during the nonfreezing period, with the highest increase reaching 35% and the lowest increase at 2.8%. Δ*f* had a negative correlation with the thickness of sediments and can be estimated by Equation (12).
(d) During the nonfreezing period, the site amplification factor for all the test sites was higher than during the freezing period, with the maximum increase reaching 38% and the minimum increase at 11%. The surface layer environment played a crucial role in the variation in the amplification factor between the frozen and unfrozen sites.

## Figures and Tables

**Table 1:** The *V_s_*_20_, *V_s_*_30_, and *h* for the test sites.

Site Number	1	2	3	4	5	6	7	8
Site Location	Harbin	Harbin	Harbin	Harbin	Harbin	Harbin	Tarim basin	Tarim basin
*h* (m)	75	45	22	23	58	62	6	11
$v_{s20}$ (m/s)	180	215	241	244	165	188	318	317
$v_{s30}$ (m/s)	205	240	260	269	194	213	558	520
Classification	C	C	C	C	C	C	B	B

**Table 2:** The predominant frequency and peak value of the horizontal and vertical Fourier spectra.

Site No.		Predominant Frequency				Peak Value			
		Unfrozen	Frozen	Δ*f_m_*	Δ*f_m_* (%)	Unfrozen	Frozen	Δ*A_m_*	Δ*A_m_* (%)
1	H	2.2	3.0	0.8	32%	17.5	12.6	4.9	28%
	V	4.9	5.0	0.1	2%	17	13	4	24%
2	H	5.0	5.6	0.6	12%	33	15	18	55%
	V	3.6	3.8	0.2	6%	25	13	12	48%
3	H	2.9	3.4	0.5	17%	12.5	10	2.5	20%
	V	6.3	6.5	0.2	3%	27.5	25	2.5	9%
4	H	3.2	4.0	0.8	25%	24	20	4	17%
	V	3.2	3.3	0.1	3%	24	20	4	17%
5	H	4.0	4.4	0.4	10%	16.5	5.2	11.3	68%
	V	3.7	4.1	0.4	11%	24	10	14	58%
6	H	2.9	3.2	0.3	10%	13.5	7.0	6.5	48%
	V	3.6	3.8	0.2	6%	8.5	8.0	0.5	6%
7	H	9.1	11.6	2.5	27%	41.5	36	5.5	13%
	V	10.2	11.2	1	10%	40	36	4	10%
8	H	6.1	8.2	2.1	34%	12.5	10	2.5	20%
	V	7.9	8.5	0.6	7.6%	14	12.5	1.5	11%

**Table 3:** Site predominant frequency in winter and summer.

Site No.	*h/m*	*f*/Hz			
		Summer	Winter	Δ*f*	Δ*f* (%)
1	75	1.2	1.24	0.04	3.3%
2	45	2.05	2.14	0.09	4.4%
3	22	3.4	3.9	0.5	14.7%
4	23	1.7	2.05	0.35	21%
5	58	1.61	1.7	0.08	5.0%
6	62	1.8	1.85	0.05	2.8%
7	6	10.2	13.8	3.6	35%
8	11	5.2	6.9	1.7	33%

**Table 4:** Site amplification factor in different seasons.

Site No.	The Ground Surface	Amplification Factor			
		Unfrozen	Frozen	Δ*A*	Δ*A*(%)
1	Soil	12.4	7.7	4.7	38%
2	Soil	12	7.5	4.5	38%
3	Soil	35	25	10	29%
4	Asphalt road	17	14	3	17%
5	Soil	14	9.7	4.3	30%
6	Concrete	13	11.5	1.5	11%
7	Soil	11	7.1	3.9	35%
8	Soil	9.1	6.4	2.7	30%

**Table 5:** $v_{s30}$ and ground types for frozen and unfrozen period (unit: m/s).

Site Number		1	2	3	4	5	6	7	8
Summer	$v_{s30}$	205	240	260	269	194	213	558	520
	type	C	C	C	C	C	C	B	B
Winter	$v_{s30}$	221	259	291	302	227	233	601	580
	type	C	C	C	C	C	C	B	B

## Data Availability

Not applicable.
